# Heterogeneous stiffness of the bone marrow microenvironment regulates the fate decision of haematopoietic stem and progenitor cells

**DOI:** 10.1111/cpr.13715

**Published:** 2024-07-09

**Authors:** Guolin Shi, Zhuo Chang, Pan Zhang, Xiaohang Zou, Xinmin Zheng, Xiru Liu, Jinxiao Yan, Huiyun Xu, Zhenhao Tian, Nu Zhang, Ning Cui, Leming Sun, Guangkui Xu, Hui Yang

**Affiliations:** ^1^ School of Life Sciences Northwestern Polytechnical University Xi'an China; ^2^ Engineering Research Center of Chinese Ministry of Education for Biological Diagnosis Treatment and Protection Technology and Equipment Xi'an China; ^3^ Center of Special Environmental Biomechanics & Biomedical Engineering Northwestern Polytechnical University Xi'an China; ^4^ Laboratory for Multiscale Mechanics and Medical Science, Department of Engineering Mechanics, State Key Laboratory for Strength and Vibration of Mechanical Structures, School of Aerospace Engineering Xi'an Jiaotong University Xi'an China; ^5^ School College of Food Science and Engineering Shaanxi University of Science and Technology Xi'an China

## Abstract

The bone marrow (BM) niches are the complex microenvironments that surround cells, providing various external stimuli to regulate a range of haematopoietic stem cell (HSC) behaviours. Recently, it has been proposed that the fate decision of HSCs is often correlated with significantly altered biophysical signals of BM niches. To thoroughly elucidate the effect of mechanical microenvironments on cell fates, we constructed 2D and 3D cell culture hydrogels using polyacrylamide to replicate the mechanical properties of heterogeneous sub‐niches, including the inherent rigidity of marrow adipose tissue (2 kPa), perivascular tissue (8 kPa) and endosteum region (35 kPa) in BM. Our observations suggest that HSCs can respond to the mechanical heterogeneity of the BM microenvironment, exhibiting diversity in cell mechanics, haematopoietic pool maintenance and differentiated lineages. Hydrogels with higher stiffness promote the preservation of long‐term repopulating HSCs (LT‐HSCs), while those with lower stiffness support multi‐potent progenitors (MPPs) viability in vitro. Furthermore, we established a comprehensive transcriptional profile of haematopoietic subpopulations to reflect the multipotency of haematopoietic stem and progenitor cells (HSPCs) that are modulated by niche‐like stiffness. Our findings demonstrate that HSPCs exhibit completely distinct downstream differentiated preferences within hydrogel systems of varying stiffness. This highlights the crucial role of tissue‐specific mechanical properties in HSC lineage decisions, which may provide innovative solutions to clinical challenges.

## Graphical abstract

HSPCs respond to the mechanical microenvironment of the BM. 3D hydrogels with higher stiffness promote LT‐HSC maintenance, whereas those with lower stiffness support MPP viability. A comprehensive transcriptional profile and fate trajectory of HSPCs suggest that niche‐like stiffness leads to differences in the components and lineage decision of heterogeneous HSPCs.

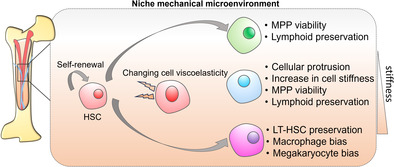

## 1 INTRODUCTION

The mechanical and confinement constraints of the 3D microenvironment have proven important for the regeneration of stem cells.^1^ Intrinsic and extrinsic mechanical forces from the cell microenvironment drive the self‐renewal, proliferation and differentiation of their residing stem cells, and promote the assembly of developing cell growth into higher‐order and functional tissue. Haematopoietic stem cells (HSCs) are at the apex of the mammalian blood system. The multipotency and self‐renewal capacities of HSCs enable them to maintain the pool size of the haematopoietic stem and progenitor cells (HSPCs) in the microenvironment throughout life. The HSPC pool comprises HSCs and differentiated haematopoietic progenitor cells (HPCs), which responsible for the balance of immune homeostasis by continuously complementing multiple types mature cells.^2^

Adult HSCs predominantly reside in the bone marrow (BM) microenvironment, commonly referred to as the BM niche. The HSCs and their functional niche, transmit biochemical and mechanical signals, enabling the integration of diverse cues that manipulate the status, position, and conduct of cells over time. Recently, the mechanical forces intersecting BM niches have been identified as central regulators of the haematopoietic programme. Interactions among cells and the inherent mechanical tension of the extracellular matrix, including matrix stiffness, topographic roughness and geometrical features, play a crucial role in the development of a functional microenvironment.^3^ Cells across various sub‐niches in the bone marrow can instantly accumulate and sense these forces triggered by external stimulations and intracellular cytoskeleton filaments. For instance, the transfer of forces in extremely supple substrates, such as elastin, is almost instantaneous and faster than biochemical signals during morphogenesis.^1^ Together with biochemical prompts, mechanical stimuli have extensive consequences for HSC quiescence, self‐renewal and lineage determination.

The mechanical properties of different sub‐niches exhibit prominent temporal and spatial heterogeneities due to the anatomical alterations of ECM. Various niche components, such as the type of ECM protein, local cell type, and spatial distribution of tissue‐specific structures, synthetically influence the heterogeneous matrix stiffness of ECM near the bone surface, vascular and adipose tissues in BM. The endosteum niches with a stiffness of approximately 40 kPa, inhabited by bone cells and abundant FN, notably differ from perivascular niches located near arteriolar pericytes with a stiffness of approximately 3 kPa. The stiffness of the central medullary region is found to be compliant with an order of magnitude lower than 1 kPa owing to adipocytes filling it.^4^, ^5^ The elasticity gradient feature plays a precise role in regulating HSC motility. For instance, a rigid matrix in the endosteal niche preserves HSCs in a dormant state, whereas perivascular niches provide an ideal environment to sustain LT‐HSCs and promote HSC proliferation.^6^, ^7^ Furthermore, the determination of HSC lineage in a manner responsive to stiffness allows for models of the BM niche to explore how cells respond to their mechanical microenvironment. Recent evidence has documented that the stiffness of constantly remodelling ECM notably determines the mechanosensitivity of the megakaryocytic lineage from HSPCs.^8^ Prevention of proplatelet production is possible by increasing the environmental stiffness from normal marrow (1.5 kPa) to malignant fibrosis (90 kPa). This requires Itgb3 to perceive FN‐coated polydimethylsiloxane substrates.^9^ However, systematic understanding of matrix stiffness within sub‐niches and the balance of haematopoiesis involving multiple immune lineages needs thorough investigation. Therefore, comprehending HSC mechanobiology and interactions with the mechanical microenvironment remains a central issue. This study focuses on the engagement of the heterogeneous mechanical properties within distinct BM sub‐niches. By creating 2D and 3D PA hydrogels that mimic their specific stiffness, we aimed to explore the crucial role of mechanical properties in governing the activity and differentiation of HSCs as well as the pool size of HSPCs. Additionally, we utilised scRNA‐seq to precisely trace the journeys of haematopoietic differentiation towards various immune lineages with high resolution, describing how the stiffness of the tissue‐specialised environment modulates the fate of HSPCs distributed in various regions. These findings indicate that matrix stiffness plays a crucial role in directing and maintaining the multipotency of HSPCs.

## 2 RESULTS

### 2.1 Construction of PA hydrogels to engineer the matrix stiffness of the bone marrow niche

**FIGURE 1:**
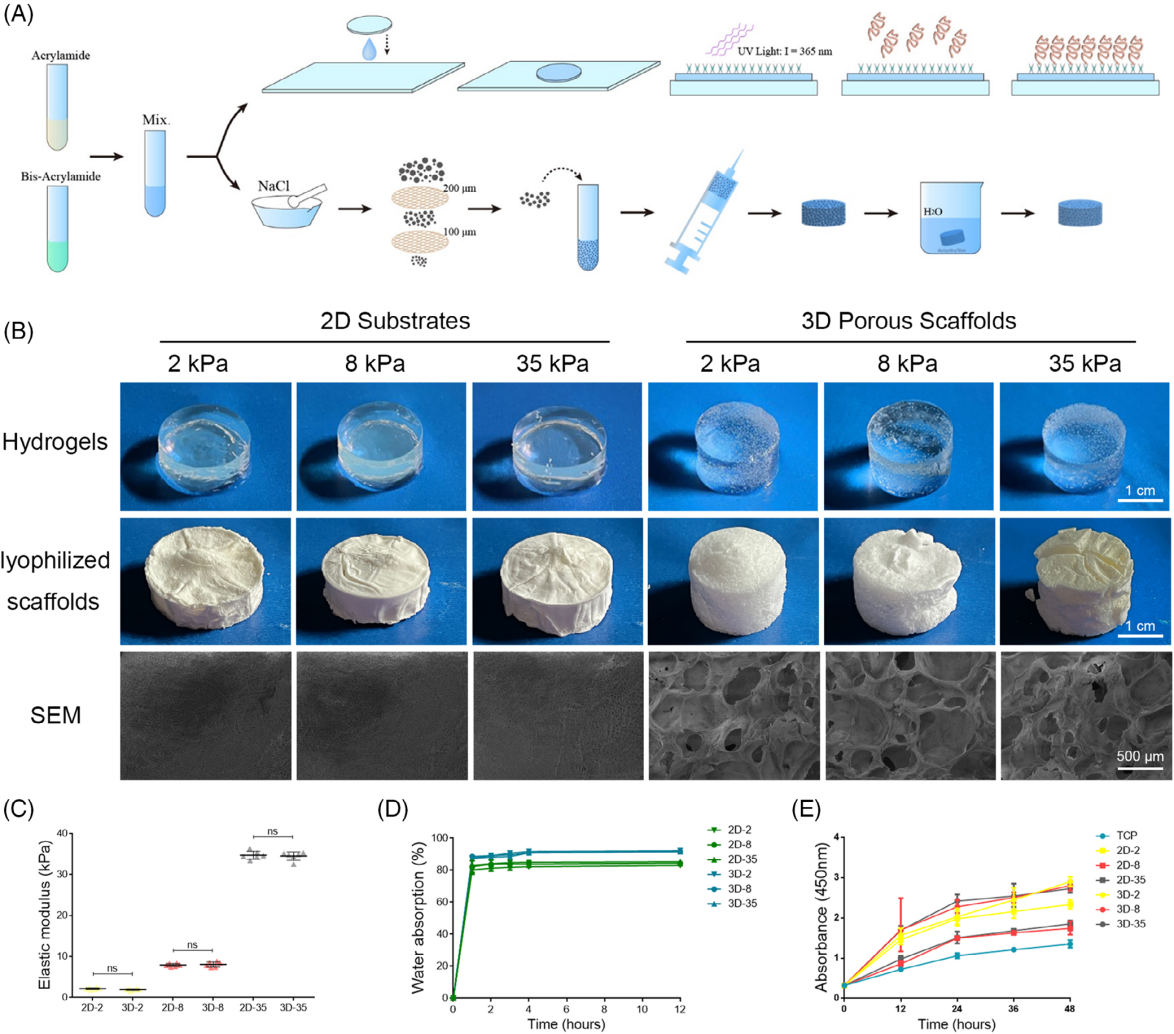
Construction and characterisation of two‐dimensional and three‐dimensional PA gels with different stiffness. (A) The fabrication process of 2D and 3D polyacrylamide hydrogels. (B) Surface morphology and SEM images of PA hydrogels and lyophilised scaffolds. (C) The Young's modulus of 2D and 3D hydrogels (*n* = 9 recipients per group from three separate experiments). (D) Water absorption of polyacrylamide hydrogels. (E) Gel stiffness influence the proliferation ability of HPCs in vitro. 2D‐2 (2D‐2 kPa). 2D‐8 (2D‐8 kPa). 2D‐35 (2D‐35 kPa). 3D‐2 (3D‐2 kPa). 3D‐8 (3D‐8 kPa). 3D‐35 (3D‐35 kPa). Three biological replicates are represented in (D) and (E). *p < 0.05; **p < 0.01; ***p < 0.001. HPC, haematopoietic progenitor cell; TCP, tissue culture plate.

Bone marrow matrix stiffness is an important mechanical regulatory parameter supporting functional HSCs. To investigate the effects of stiffness on HSC expansion and differentiation, 2D and 3D scaffolds with niche‐like mechanical properties were prepared using a stiffness‐tunable polyacrylamide gelation assembly (Figure 1A). By adding NaCl particles to the liquid prior to gelation, the porous structure of the 3D systems was created, allowing the diffusion of water and growth factors (Figure 1B). Mouse HSPCs were cultured between top and bottom PA gels of different Young's modulus (E′). The soft substrates (E′ = 2.12 ± 0.33 kPa), moderate substrates (E′ = 8.12 ± 0.62 kPa) and hard substrates (E′ = 35.12 ± 0.41 kPa) represented the natural elasticity of different sub‐niches, including adipose tissue, vessel wall and endosteum in BM (Figure 1C). The porous structure improved the water absorption capacity of the hydrogels (Figure 1D). The cytocompatibility of the PA gels was demonstrated in Figure 1E. Compared to the conventional liquid culture on tissue culture plate (TCP), the hydrogel systems significantly motivated the cell proliferation activity, especially the 3D systems.

### 2.2 The impact of matrix stiffness on cell morphology and mechanics

**FIGURE 2:**
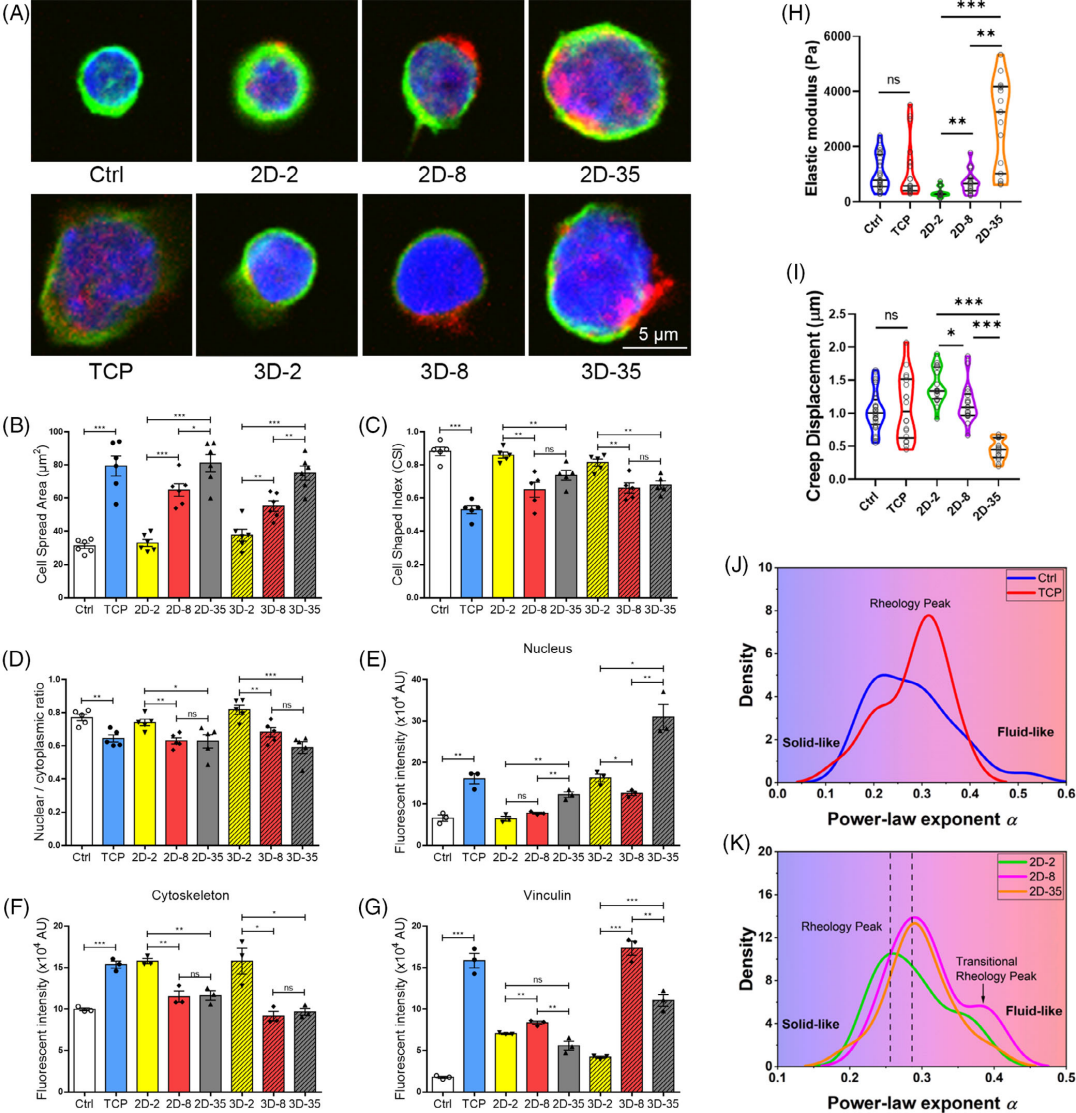
Mechanical characteristics of HPCs on different substrates. (A) Morphological changes of cell shape captured after staining, quantified as (B) cell spread area (μm^2^) and (C) CSI. (D) Nuclear/cytoplasmic ratio of stained cells. (E) Quantification of stained nucleus. (F) Quantification of stained cytoskeleton. (G) Quantification of stained vinculin (*n* = 3–5 recipients per group from three separate experiments). (H) The static elastic modulus and (I) viscous creep displacement of cells on varying substrates (*n* = 10 recipients per group from three separate experiments). (J–K) The distribution profiles of power‐law exponent of cells on TCP and gel substrate with variable stiffness. Ctrl (freshly sorted HPCs). 2D‐2 (2D‐2 kPa). 2D‐8 (2D‐8 kPa). 2D‐35 (2D‐35 kPa). 3D‐2 (3D‐2 kPa). 3D‐8 (3D‐8 kPa). 3D‐35 (3D‐35 kPa). Green, F‐actin; blue, cell nucleus; red, focal adhesion. *p < 0.05; **p < 0.01; ***p < 0.001. CSI, cell‐shaped index; HPC, haematopoietic progenitor cell; TCP, tissue culture plate.

We examined how matrix stiffness influences the morphology and mechanical response of haematopoietic cells. To recreate the diverse mechanical situations in vivo, cells were grown on gels with three levels of stiffness: 2 kPa, 8 kPa and 35 kPa. Confocal graphs revealed that mechanistically distinct microenvironments had a significant impact on the shape of HPC spreading (Figure 2A–G). Compared to freshly sorted cells, the fluorescence signal representing focal adhesion can be observed on the cells cultured in vitro, which was significantly enhanced on both the moderately stiff at 8 kPa and the stiff at 35 kPa (Figure 2A). These data suggest that matrix stiffening‐induced focal adhesion formation is involved in the interaction of cells with their mechanical environment. The morphological variations of HPCs were determined using cell spreading area and cell‐shaped index (CSI). In contrast to the freshly sorted cells, the cells on the stiff gel surface (35 kPa) exhibited maximal spreading, while on the softer substrate (2 kPa and 8 kPa), the cells remained in a more rounded shape (Figure 2B,C). Similarly, cell spreading in 3D PA hydrogels increased as the stiffness increased, with smaller cells but increased nuclear/cytoplasmic (N/C) ratio observed in the 2 kPa matrix. The lowest N/C ratios observed in the 3D‐35 kPa group (approximately 0.8) demonstrated more intranuclear events activated on stiff substrate (Figure 2D). In addition, cells in 3D gels of 35 kPa showed higher fluorescence intensity of the nucleus (Figure 2E), whereas cells in 2 kPa gels showed higher fluorescence intensity of the cytoskeleton (Figure 2F). In addition, cells in 8 kPa gels showed higher fluorescence intensity of vinculin (Figure 2G).

To examine how cell mechanics, particularly cell viscoelasticity, respond to changing mechanical environments, HPCs were extracted from the gels and scrutinised using AFM. Cells isolated freshly and cultured for 2 h acted as the control (Ctrl group). The AFM technique was used to scrutinise the static elastic stiffness and dynamic creep response of cells from different groups (Figure 2H–K). Importantly, the results demonstrate that the overnight growth of cells on a rigid TCP substrate has no significant effect on their elastic modulus (Ctrl: 1059.54 ± 634.64 Pa; TCP:1125.36 ± 1049.00 Pa, *p* = 0.49). Conversely, when cultivated on more rigid gel substrates, cell stiffness greatly increases. Interestingly, cells on soft gels (2D‐2: 345.09 ± 169.11 Pa) display a softer phenotype, whereas their elastic modulus increased two‐fold and eight‐fold on the moderate and hard substrates (2D‐8: 703.91 ± 400.18 Pa; 2D‐35: 2891.71 ± 1623.96 Pa, Figure 2H). It should be noticed that cells on the hard gel substrate are characterised by a higher stiffness than those on TCP substrates. Subsequently, we examined the dynamic mechanical properties of cells on various substrates. During the force clamping and a dwell period of 10 s, we quantified the creep displacement of samples, serving as an indicator of time‐dependent dynamic creep deformation in cells (Figure 2I). We observed that an overnight culture has insignificant effects on the viscous creep of cells (Ctrl: 1039.65 ± 305.25 nm; TCP: 1086.73 ± 483.63 nm, *p* = 0.63). When seeded onto gel substrates, cells on a hard substrate demonstrate minimal viscous creep, under a given load of 800 pN. In contrast, cells on a soft substrate exhibit the most prominent viscous creep, as shown by measurements of 2D‐2 (1411.51 ± 259.69 nm), 2D‐8 (1152.35 ± 295.64 nm) and 2D‐35 (448.52 ± 152.88 nm). In contrast, cells on a soft substrate exhibit the most prominent viscous creep, as shown by measurements of 2D‐2 (1411.51 ± 259.69 nm), 2D‐8 (1152.35 ± 295.64 nm) and 2D‐35 (448.52 ± 152.88 nm). Taking the static and dynamic mechanical results collectively, a significant rise in mechanical heterogeneity among cells is evident, highlighted by the coefficient of variation (CV), as the gel substrate stiffness increases (elastic stiffness: 49.00%, 56.85% and 56.16%; viscous creep: 18.40%, 25.66%, 34.09% for cells on gels with stiffness of 2 kPa, 8 kPa and 35 kPa respectively). More significantly, cells on more rigid substrates (8 kPa and 35 kPa) experience higher alterations in their viscous creep feature compared to their static elastic rigidity. This leads to an increase in CV of 14.6% for elastic rigidity and 85.22% for viscous creep during the transition from 2 kPa to 35 kPa.

In addition, we define the time‐dependent creep compliance *J*(*t*) as the ratio of cell strain *ε*(*t*) to applied stress *σ*(*t*). Plotting *J*(*t*) as a function of time in a log–log scale reveals a consistent power‐law rheology with power‐law exponents (*α*) falling within the range of 0.1–0.5, consistent with previous studies (Figure 2J). The power‐law exponent *α* = 0 and *α* = 1 represent a purely elastic solid and viscous fluid, respectively, which effectively capture the viscoelastic properties of the cells. Despite no statistical differences are observed in the *α* among cells on varying substrates (*p* = 0.64), subtle alterations are found in the distribution profiles and rheology peaks of *α* among these substrates. Following overnight culture on glass substrates, the rheology of cells shifts towards a more fluid‐like characteristic (rheology peak: ~0.2 for Ctrl and ~0.35 for TCP). We also notice a series of delicate changes in the rheology peaks and power‐law distribution profiles of the cells on various substrates (Figure 2K). Remarkably, we find that the rheology peak of cells on soft substrate shift from ~0.25 to ~0.3 when cultured on harder gels. Additionally, a dual rheology peaks emerge, with ~0.3 for the low rheology peak and ~0.4 for the high rheology peak. The low rheology peak for soft and moderate hard gels are excellently overlapped, while the high rheology peak for the 2D‐8 groups might characterise the transitional state of cells on moderate hard gel. Taken together, our findings demonstrate that mechanical characteristics of HPCs, including static elastic stiffness and dynamic viscous creep, as well as their distribution profiles and heterogeneity, are altered on substrates of different stiffness.

### 2.3 The BM niche‐like stiffness regulates the viabilities of HSPC subgroups

**FIGURE 3:**
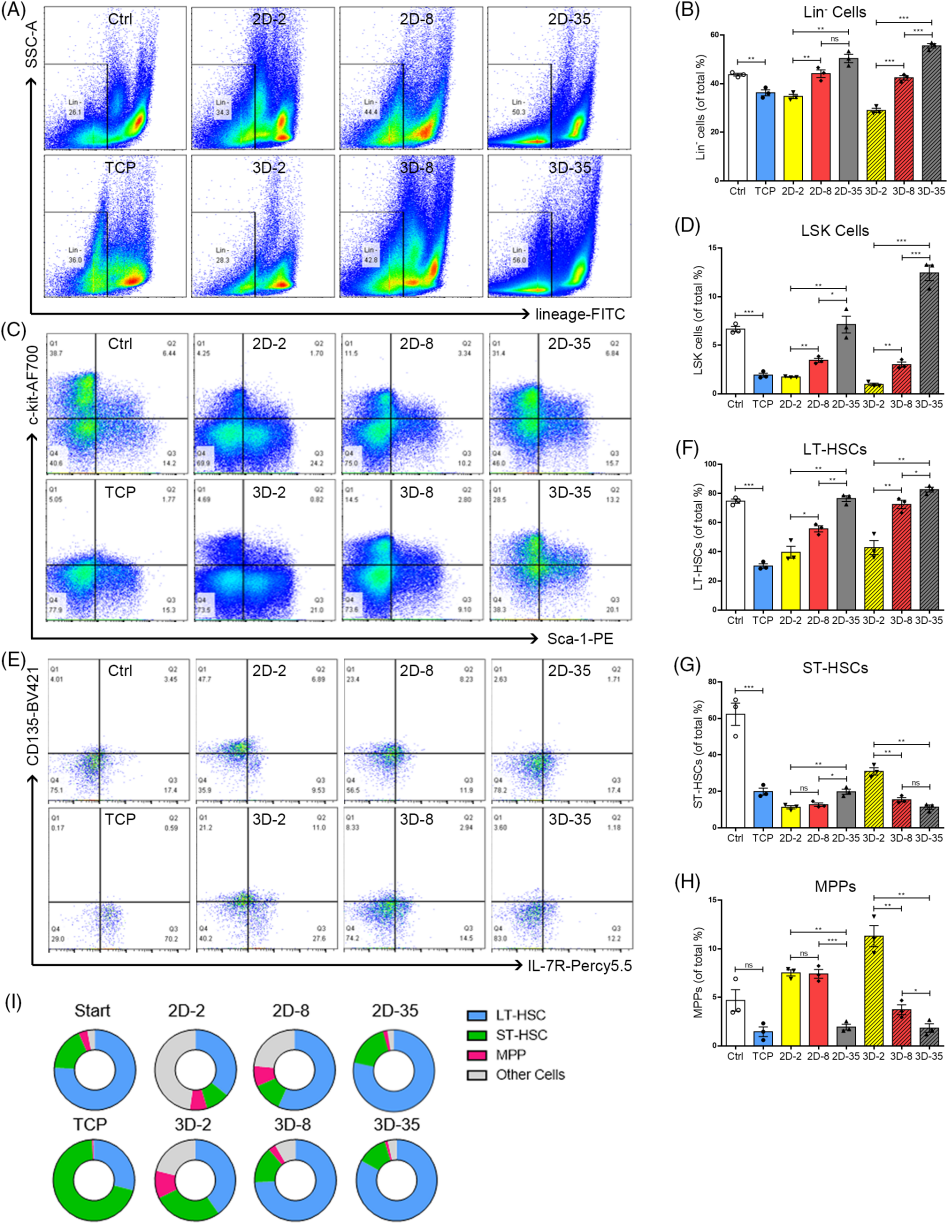
The effect of matrix‐stiffness on the fractions of stem/progenitor subgroups in HSC pool. Representative flow cytometry results indicate the percentages of (A) Lin^−^ cell percentages, (C) LSK (Lin^−^Sca‐1^+^c‐kit^+^) cells and (E) LT‐HSCs (CD34^−^CD135^−^LSK). Comparative analysis for percentages of (B) Lin^−^ cells, (D) LSK cells, (F) LT‐HSCs, (G) ST‐HSCs, (H) MPPs on the surface of 2D gels or inside 3D scaffolds (*n* = 3 recipients per group from three separate experiments). (I) Summary of HSPC proportions on the surface of 2D gels and inside 3D scaffolds. HSC, haematopoietic stem cell; HSPC, haematopoietic stem and progenitor cell; MPPs, multi‐potent progenitors; ST‐HSCs, short‐term repopulating HSCs.

BM environments provide mechanical signals to retain HSC pools and affect the cell fate decisions of quiescence, self‐renewal and differentiation. To establish the regulatory role of tissue‐associated stiffness on HSPCs residing in spatially distinct sub‐niches, we analysed the proportions of different HSPC subsets on substrates ranging from 2 kPa to 8 kPa to 35 kPa under varying dimensional conditions. Freshly isolated HSPCs and HCPs were cultured on conventional TCP plates for evaluation. The observed changes in the percentages of LSKs (Lin^−^Sca‐1^+^c‐kit^+^ cells) and MPPs (multi‐potent progenitors, CD135^+^CD34^+^LSK) indicate the rigidity‐dependent characteristics of haematopoietic progenitors in their respective niches (Figure 3). The rigid 35 kPa surfaces were effective in preserving the vitality of Lin^−^ progenitors (Figure 3A,B), LSK (Figure 3C,D) and LT‐HSCs (CD135^−^CD34^−^LSK, Figure 3E,F), as indicated by their highest percentages on 2D and 3D gels. Notably, the percentages of LSK cells increased more when subjected to high confinement and rigid substrates of 35 kPa than when cultured on compliant hydrogels of 2 kPa and 8 kPa (Figure 3D). Conversely, the number ratios of both MPPs and short‐term repopulating HSCs (ST‐HSCs, CD135^−^CD34^+^LSK) to total progenitor cells significantly decreased under 35 kPa hydrogels (Figure 3G,H). The higher MPP ratio on the 2 kPa matrix implies that perivascular ECM favours more differentiated HPCs over LT‐HSCs (Figure 3H). On the other hand, when the substrate elasticity was moderately stiff at 8 kPa, there was a quick reduction in MPPs activities for the cells confined within the 3D matrix, as compared to those in 2D settings (Figure 3I). Hydrogels that imitate the mechanical features of marrow sinusoids have been verified to be appropriate for conserving MPP percentages. Moreover, an enhanced imposition of spatial constraint negatively affects the preservation of MPPs.

### 2.4 The BM niche‐like stiffness regulates the differentiation potentials of HSPCs

**FIGURE 4:**
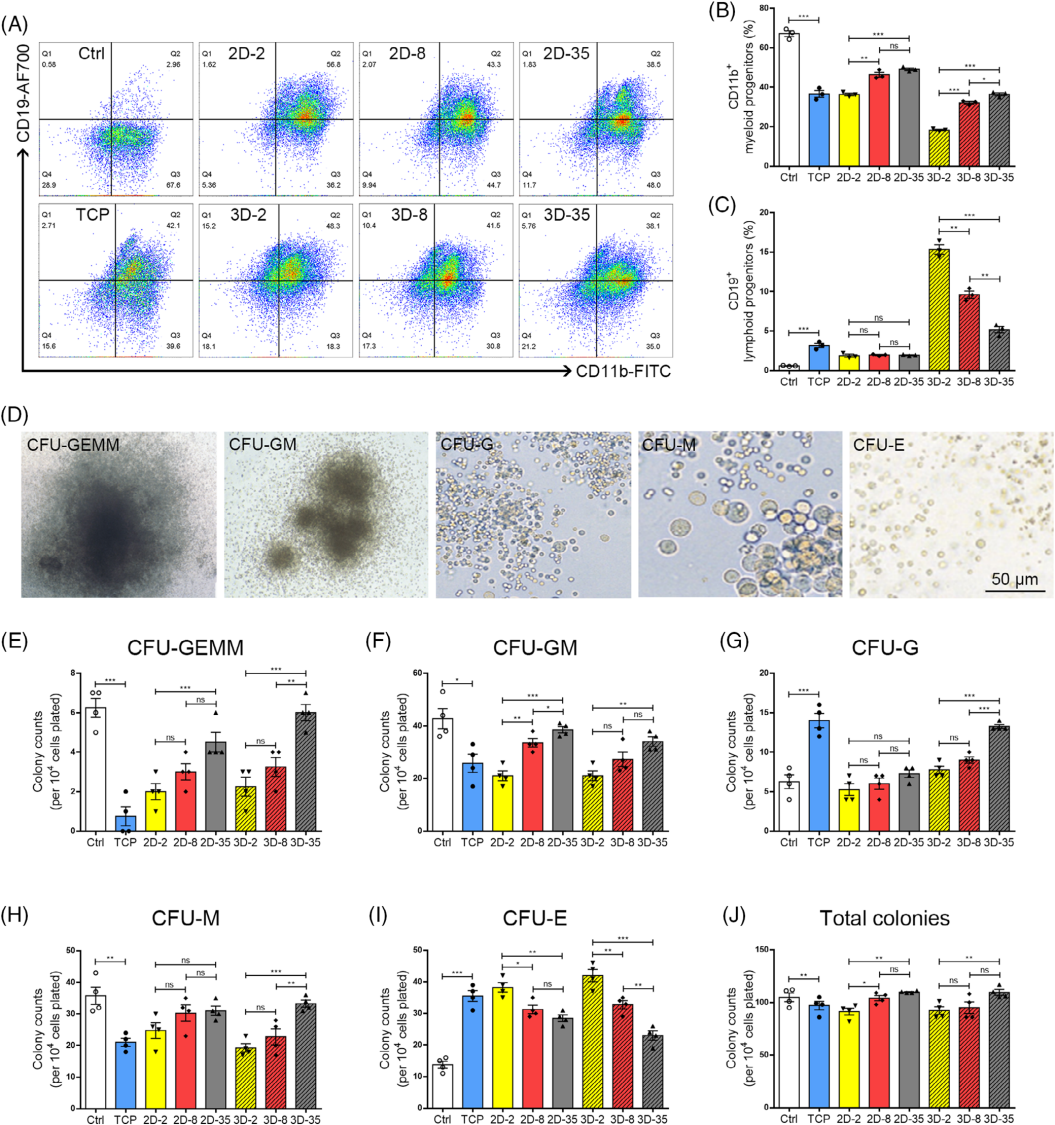
Matrix‐stiffness regulate the differentiation potentials of HPCs. (A) Flow cytometry photographs reflected the effects of niche‐like stiffness on the percentages of CD11b^+^ myeloid cells and CD19^+^ lymphoid cells. Comparative analysis for percentages of (B) myeloid cells and (C) lymphoid cells (*n* = 3 recipients per group from three separate experiments). (D) Morphonology of distinct CFU‐colonies after magnifying using an optical microscope. Scale bar: 50 μm. Colony formation ability signifies the potential of HPCs differentiating into different stages of myeloid and erythroid lineages, including (E) CFU‐GEMM (colony‐forming unit‐granulocyte, erythrocyte, macrophage, megakaryocyte), (F) CFU‐GM (colony‐forming unit‐granulocyte, macrophage), (G) CFU‐G (colony‐forming unit‐granulocyte), (H) CFU‐M (colony‐forming unit‐macrophage), (I) CFU‐E (colony‐forming unit‐erythroid) and (J) total colonies (*n* = 3 recipients per group from three separate experiments). HPC, haematopoietic progenitor cell.

In order to determine whether variations in 3D environment stiffness affect the differentiation capabilities of HPC, flow cytometry and colony‐forming unit (CFU) assays were conducted. The commitment of HPC to lymphoid and myoid lineages was demonstrated by CD19^+^ and C11b^+^ cells, respectively^10^ (Figure 4). Following 24 h of 3D‐culture, hydrogels with 2 kPa and 8 kPa compliance were found to excel in preserving lymphoid cell percentages (Figure 4A,B). The differentiation of HPCs towards CD11b^+^ myeloid cells was enhanced in stiffer hydrogels with a modulus of 35 kPa, while the proportion of CD19^+^ lymphoid cells significantly decreased in the stiff gels (Figure 4C). These findings imply that the increased matrix stiffness modulates the specialised capacities of HPCs in 3D culture systems towards the myeloid and lymphoid lineages.

Meanwhile, we examined the colonies formation of multi‐potential progenitor cells (CFU‐GEMM), granulocyte and macrophage progenitor cells (CFU‐GM), macrophages (CFU‐M), granulocytes (CFU‐G) and erythroid progenitor cells (BFU‐E). The different types and quantities of these units demonstrated varying specialisation capabilities towards their respective haematopoietic lineage commitments, following the initial cell culture using hydrogels (Figure 4D). Hydrogels allowed for the preservation of HPC differentiation potential to CFU‐GEMM colonies, in contrast to TCP plates. The counts of CFU‐GEMM were observed to increase alongside the stiffness of hydrogels, both when used within 3D gels and when cultured on top of 2D gels (Figure 4E). The colony units derived from cells within 3D gels of 35 kPa indicate that the stiffer matrix enhances the primitive capabilities of earliest progenitor cells, possibly due to improved viabilities of LT‐HSC and LSK cells, as depicted in Figure 3. HPCs cultured on relatively stiff gels (35 kPa) also exhibited a greater number of CFU‐GM colonies, which were identified as having the potential ability to generate early stages of myoid lineage. In comparison to the gels with 2 kPa stiffness, the HPCs cultured on the intermediate soft substrates (8 kPa) preserved the differentiation of early myoid progenitors under 2D conditions (Figure 4F). Colony formation of granulocytes and macrophages can reflect late myeloid events. Cells within stiffer gels (35 kPa) exhibited more CFU‐M (Figure 4G), CFU‐G (Figure 4H) colony units, and total colonies (Figure 4J) than those cultured on soft gels. This finding further confirms the promotion of myeloid specification by increased stiffness, consistent with Harley's previous research.^4^ Compared to 3D gels with a stiffness of 35 kPa, cells cultured on TCP show a greater potential for differentiation towards CFU‐G. The comparison of CFU‐E proliferation highlighted a favourable influence of stiffer gels in supporting the differential of HPCs into erythroid cells (Figure 4I). These data strongly indicate that changes in matrix stiffness significantly impact the differentiation potential of HSPCs, particularly in the generation of early multi‐potential progenitor cells and early myeloid progenitors.

### 2.5 Single‐cell transcriptomic profiling highlights the HSPC heterogeneity regulated by 3D hydrogels

**FIGURE 5:**
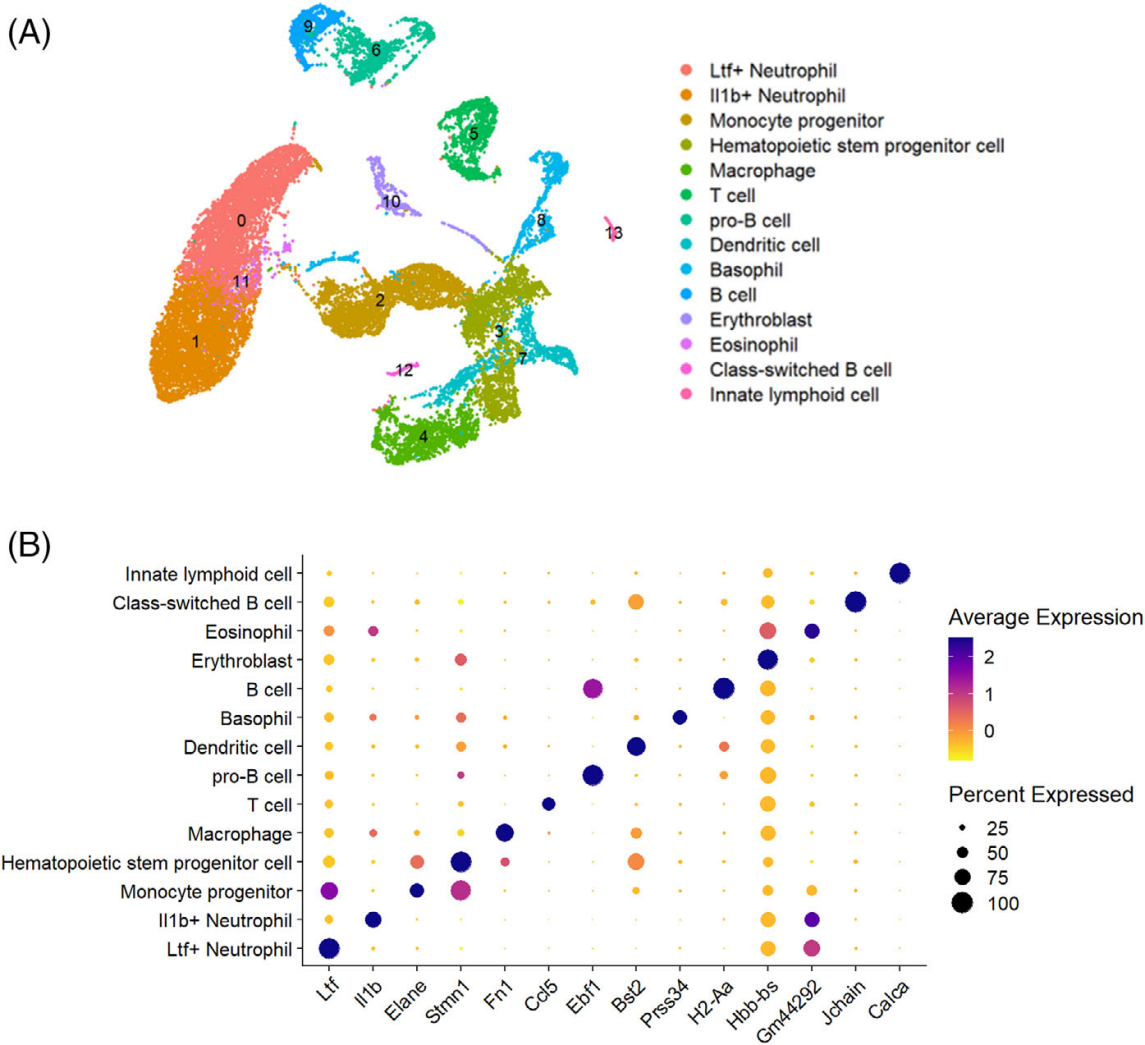
The heterogeneous haematopoietic subsets derived from 3D hydrogel scaffolds by single‐cell resolution. (A) Uniform manifold approximation and projection (UMAP) visualisation of HPCs, coloured by cluster‐specialised annotation. A total of 14 distinct clusters were defined with Unbiased Seurat clustering method. (B) Bubble chart exhibiting distinct expression levels of the selected marker genes in each cluster. HPC, haematopoietic progenitor cell.

Single‐cell RNA sequencing offers a valuable technique to quantify expression variability among individual cells, particularly for heterogeneous cellular states. The use of 3D platforms allows for the replication of in vivo phenotypes that may be disregarded in conventional 2D cultures. To enhance our comprehension of HSPC fate determination regulated by 3D mechanical microenvironment at a high resolution, we employed scRNA‐seq to examine transcription activity in 3D‐cultured HSPCs. Additionally, we monitored their dynamic developmental trajectories within 3D hydrogels of varying stiffness that simulate different niches. After culturing LSK HPCs in 3D porous scaffolds with varying stiffness (2 kPa and 35 kPa), gene expression was examined at a single cell level using the 10xGenomics Chromium platform. Data was analysed downstream with the CellRanger package. The cell‐gene matrix underwent the standard Seurat procedures, which included an initial quality control assessment based on cell filtering setup and principal component analysis. For subsequent analysis, 30 principal components (PCs) were selected with a 0.2 resolution. Consequently, a dataset of 24,640 signature genes was composed, comprising of 6727 filtered cells on gels of 2 kPa, 15,544 cells on gels of 35 kPa, and 5734 freshly sorted HSPCs. The cells have been categorised into 14 clusters based on their variable gene expression profiles. The unique clusters are visualised through uniform manifold approximation and projection (UMAP) plots, as depicted in Figure 5A. To annotate each cluster, we referenced the maker gene database for immune lineages (CellKb IMMUNE: https://www.cellkb.com/ct_search_back) and previously published markers of haematopoietic cells, using their corresponding top 10 differentially expressed genes (DEGs).

UMAP plots presented different haematopoietic commitments from a merged cell dataset: (1) HSPCs characterised by the fingerprint genes of Macroh2a1, Stmn1, and Cd34; (2) Monocytes exhibiting a high expression level of Elane; (3) Two distinct types of neutrophils expressing markers of Ltf and Il1b, respectively. (4) Basophils highly express markers Prss34 and Mcpt8. (5) Eosinophils have a high expression level of mt‐Rnr2 and mt‐Rnr1. (6) Macrophages are recognised for distinctively expressing Fn1 and S100a4. (7) Erythroblasts are characterised by the markers of Hbb‐bs. (8) Dendritic cells are recognised for distinctively expressing Bst2. (9) Innate lymphoid cells are distinguished by the distinct expression of Calca. (10) T cells highly express markers Ccl5 and Ms4a4b. (11) Pro B cells are characterised by markers of Ebf1 and Cd79b. Class‐switched B cells are characterised by markers of Igha and Jchain. The downstream B cells exhibit a high expression level of Matrix metallopeptidase Ebf1 and H2‐Aa (Figure 5B, Table S1).

### 2.6 Changing 3D environmental stiffness leads to differences in the components and lineage decision of heterogenous HSPCs

**FIGURE 6:**
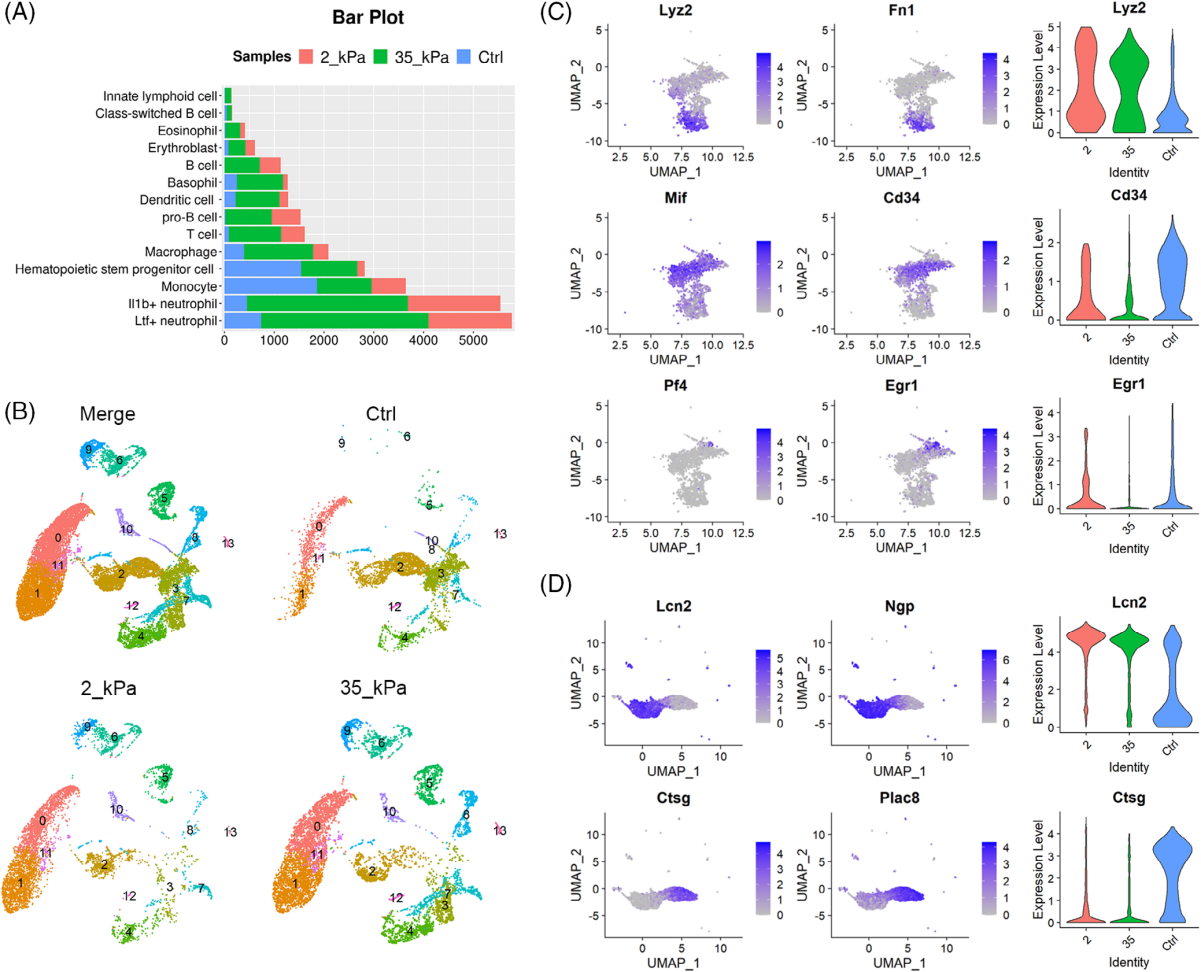
3D mechanical microenvironment leads to differences in the single‐cell landscape of haematopoietic cell populations. (A) Bar plot of the distributions of the cell count of each cluster in freshly sorted HPCs (Ctrl) and cells sorted from 3D hydrogels with different stiffness (2 kPa and 35 kPa). (B) UMAP vitalisation of relative position of successfully classified haematopoietic clusters in Ctrl, 2 kPa and 35 kPa group, respectively. The number of HSPCs (cluster 3) and Monocytes (cluster 2) significantly reduced after cultured on 3D hydrogels in vitro, in contrast to increased member of neutrophils (cluster 0/1), eosinophils (cluster 11), erythroblasts (cluster 12) and lymphoid cells (cluster 5/6/9). Higher environmental stiffness to support distinct haematopoietic populations in vitro. (C) UMAPs colour‐coded for expression (grey to blue) of HSC markers and progenitor markers to dissect the heterogeneity of HSPCs (left). Violin plots of differently expressed cell‐type markers in each subcluster. Lyz2, CD34 and Egr1 indicate haematopoietic lineages of macrophage, HSC and megakaryocyte, respectively (right). (D) UMAPs colour‐coded for expression (grey to blue) of monocyte‐associated markers to distinguish the different subclusters in monocyte cluster 2 (left). Violin plots of differently expressed monocyte markers, Lcn2 and Ctsg, in each subcluster (right). HPC, haematopoietic progenitor cell; HSC, haematopoietic stem cell; HSPC, haematopoietic stem and progenitor cell; UMAP, uniform manifold approximation and projection.

The analysis of the percentage of each cell cluster reveals a distinct distribution in three datasets (Figure 6). HSPCs (Cluster 3) and Monocytes (Cluster 2) predominated among freshly sorted cells. Culturing cells with hydrogels promoted lineage differentiation in contrast to freshly sorted HPCs, resulting in neutrophils, erythroblasts and lymphoid cells, including T cells, pro B cells and B cells (5, 6, 9). The significant rise in the number of cluster 6 and cluster 9 identified in both hydrogel groups suggests that the 3D environment can promote B cell specification (Figure 6A). When compared to HPCs on softer hydrogels with a 2 kPa Young's modulus, stiffer 3D hydrogels with a Young's modulus of 35 kPa demonstrate the ability to effectively maintain these haematopoietic populations during in vitro culture (Figure 6B).

The multi‐lineage potential of HSC is contingent on the varied transcriptomic and functional lineage bias of their subtypes.^11^ To effectively elicit details of pre‐determined behaviour resulting from matrix mechanic features, gene characteristics of HSPCs and Monocyte clusters from the extensive dataset were subsequently chosen for further categorisation (Figure 6C,D). Using the UMAP algorithm, three distinct subpopulations of HSPCs were identified. The subset exhibiting high expression of HSC markers Mif and Cd34 was predominantly identified among freshly sorted cells. The expression of Pf3 and Egr1 suggested the presence of megakaryocyte progenitor cells within the HSPC population. Upon culturing with hydrogels of 35 kPa, a subset of cells that simultaneously expressed the monocyte marker Fn1 and macrophage marker Lyz2 emerged in the HSPC, indicating a lineage towards macrophages that has been classified as cluster 4 (Figure 6C). This highlights that the benefits of rigid substrates mimic the stiffness of the endosteum region, which is crucial for maintaining early haematopoietic progenitors. Cluster 2 was subdivided into two separate subclusters of cells associated with monocytes. One of these subclusters was identified by the expression of the Lcn2 and Ngp markers, while the other was found to be relatively similar to the previously labelled HSPC population and displayed noteworthy expression of the markers Ctsg and Plac8 associated with HPCs (Figure 6D). Compared to HPCs sorted from the native bone marrow, cells cultured on 3D hydrogels exhibited a decreased subpopulation of HPCs in the monocyte cluster, particularly on compliant gels. The single‐cell transcriptomic signatures for HSPCs validate the analogue inclination demonstrated in CFU‐forming assays and additionally disclose that substrates with heightened elasticity can foster macrophage bias, even during the early stages of haematopoietic development.

**FIGURE 7:**
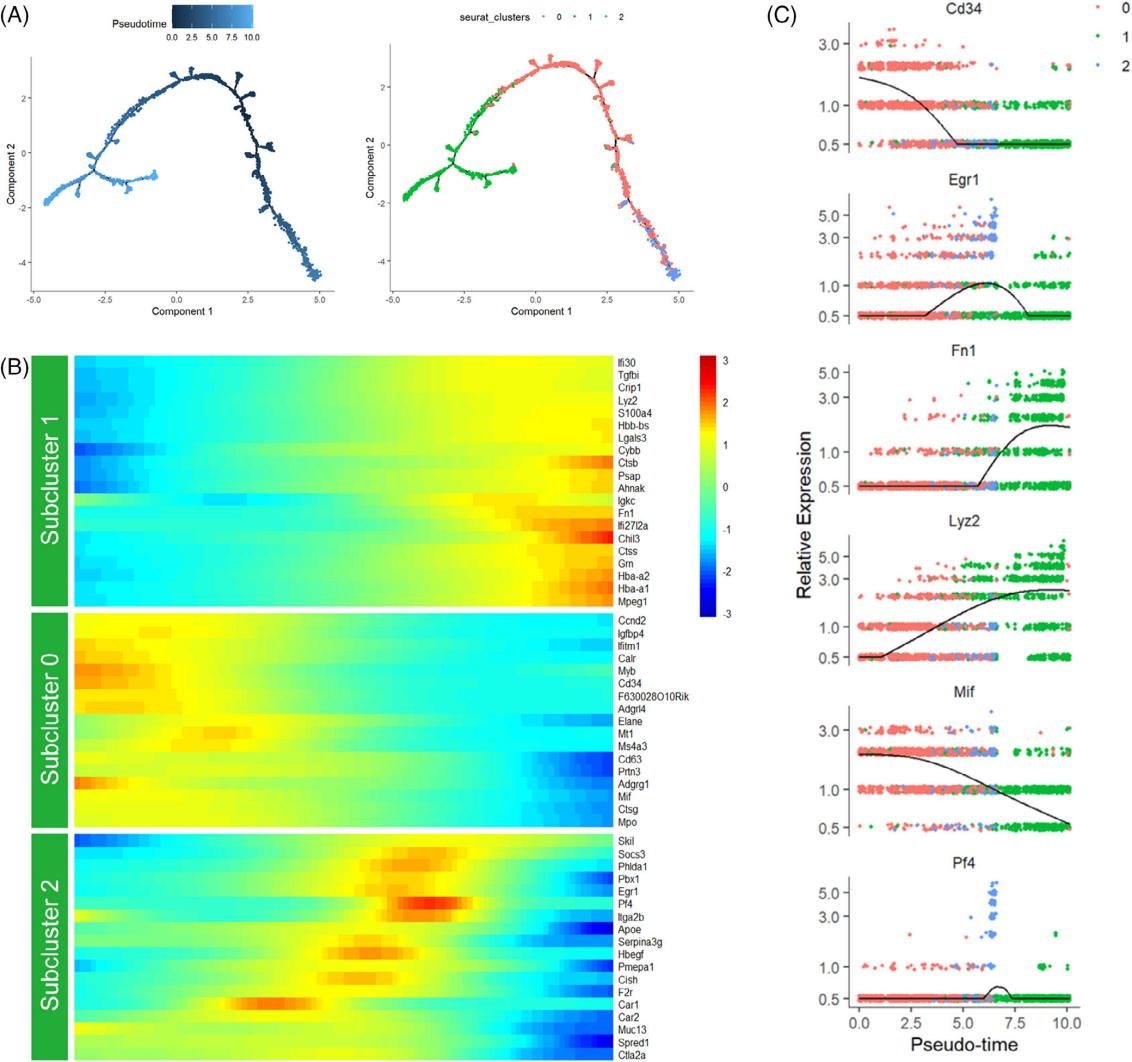
Dynamic developmental trajectories of previously identified subpopulations within HSPCs. (A) Monocle analysis predicts pseudotime line (left) and developmental hierarchies (right) for HSCs (subcluster 0), macrophage progenitors (subcluster 1) and megakaryocyte progenitors (subcluster 2). (B) Heatmap displaying the scaled gene expression of DEGs involved in the dynamic lineage differentiation from pre‐branch of HSCs along the trajectory to haematopoietic branches. (C) The expression levels of specific representative DEGs along pseudotime trajectory. Cd34 and Mif were transiently expressed in HSCs, while the expressed curve of Fn1 and Lyz2 raise from the centre to the right margin. DEG, differentially expressed genes; HSC, haematopoietic stem cell; HSPC, haematopoietic stem and progenitor cell.

Next, we performed dynamic trajectory analysis to elucidate the internal maturation cascades of homogeneous HSPCs in cluster 3. The monocle algorithm (Figure 7) was used to predict the position of each cell on a differential timeline. We can more accurately determine the developmentally hierarchical relationship from multipotent HSCs to the various haematopoietic subsets that contribute to the altered lineage output. The subset of HSCs appeared at the beginning of the pseudotime trajectory, reflecting the highest ‘source’ state in cell specification line. The subset of macrophage progenitors occupied the branch extending from the original HSC subset, while megakaryocyte progenitors appeared at another trajectory branch (Figure 7A). Cells situated towards the end of one branch reflected a relatively later developmental stage and shared common upriver progenitors. Regarding the amount of HSC subgroups in the original BM, the subsequent pseudotime sequence of the macrophage precursor branches revealed the transmission of cell state through rigid matrix stiffness in vitro. The significant alterations in lineage‐specific gene expression patterns were consistent with the pseudotime coordinates (Figure 7B,C), which corresponded to the distribution of the three identified subpopulations in the UMAP plots. The upregulation of Lyz2 and Fn1 expression, subsequent to a decrease in Cd34 and Mif, indicated the transition of HSC to the macrophage lineage (Figure 7C). ScRNA‐seq proved a useful method in characterising the molecular changes of HSCs originating from distinct mechanical microenvironments. These findings highlight the impact of mechanical properties of subniches on the alteration in equilibrium between predisposed HSC subsets. Based on the pseudotime transcriptional dynamics at a single level, we have shown the inherent BM HSPC hierarchy consisting of macrophage‐biased and megakaryocyte‐biased progenitors. The endosteum region with higher stiffness can increase the molecular priming to macrophage differentiation.

### 2.7 3D mechanical microenvironment introduces functionally alteration processes during HSPC differentiation

**FIGURE 8:**
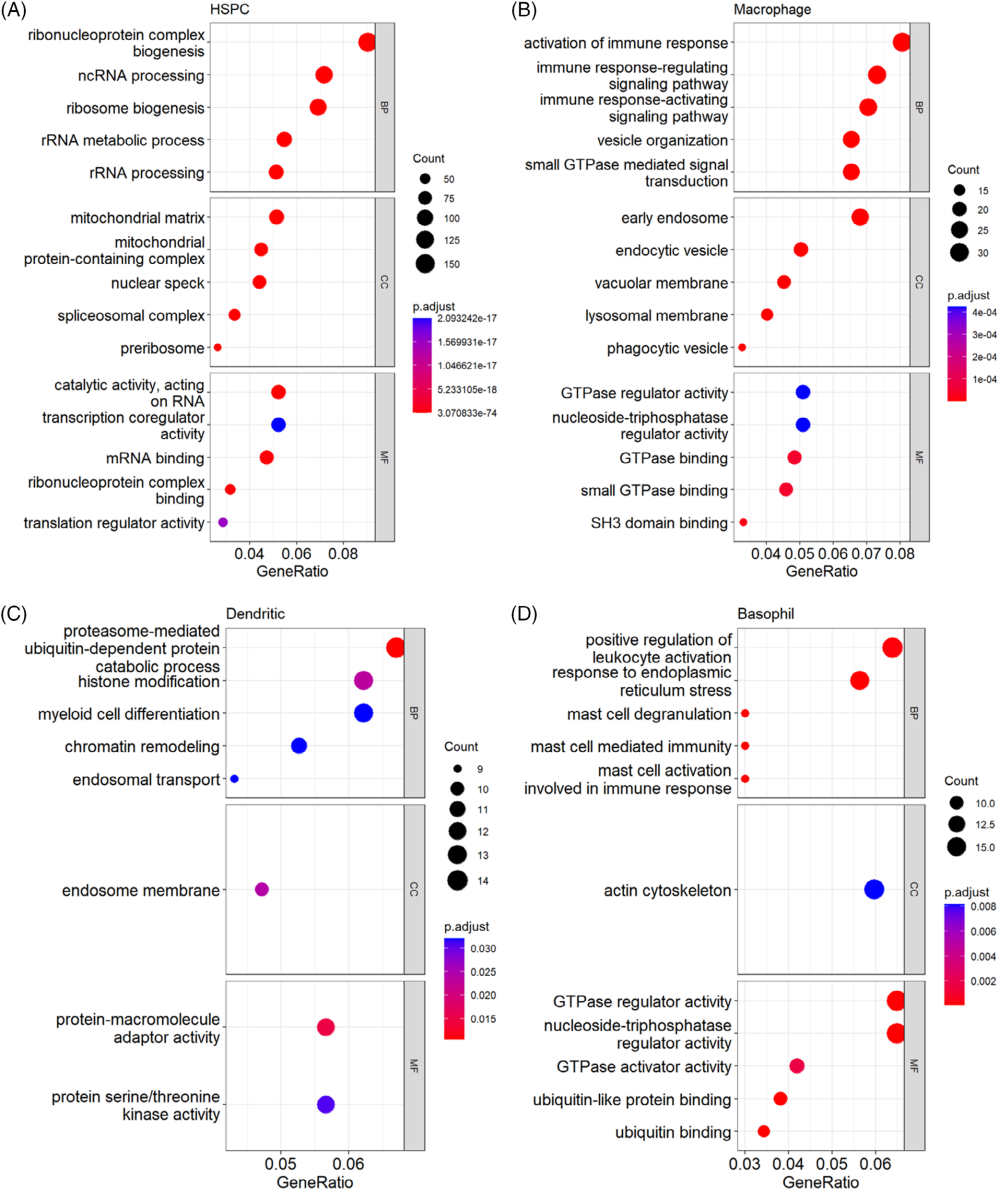
GO analysis of highly expressed genes enriched to (A) HSPC, (B) macrophages, (C) dendritic cell and (D) basophil provides unique functional processes identified for each population. HSPC, haematopoietic stem and progenitor cell.

To identify functional processes that are relevant to the clusters and are regulated by matrix stiffness, the DEGs belonging to myeloid clusters of interest were analysed using GO (Gene Ontology) functional enrichment analysis. This analysis offers a greater insight into the physiological processes that are related to cell types of previously identified haematopoietic populations, with regards to three distinct categories including biological process (BP), molecular function (MF) and cellular component (CC). GO terms enriched in populations of HSPC, macrophage, dendritic cell and basophil reflect the dynamic processes of HPC development inspired by 3D hydrogels (Figure 8). The top‐level GO terms of HSPC were significantly relevant to the classifications of ‘ribonucleoprotein complex biogenesis’, ‘mitochondrial matrix’ and ‘catalytic activity, acting on RNA’, indicating an activated progenitor pool. The macrophage cluster was primarily enriched with GO terms such as ‘activation of immune response’, ‘early endosome’ and ‘SH3 domain binding’, as demonstrated in Figure 8A. Furthermore, the DEGs expressed in macrophages displayed significant correlation with function categories associated with GTPase binding and activity (Figure 8B), which participate in coordinating the organisation of the actin cytoskeleton.^12^ In conjunction with the increased cell numbers of 4, 7 and 8 in stiff hydrogels, the function enrichment suggests that the stiff endosteum sub‐niche is conducive to driving the formation of myeloid cells such as macrophages, dendritic cells, and basophils, with highly expressed DEGs commonly involved in immune functions (Figure 8C,D).

**FIGURE 9:**
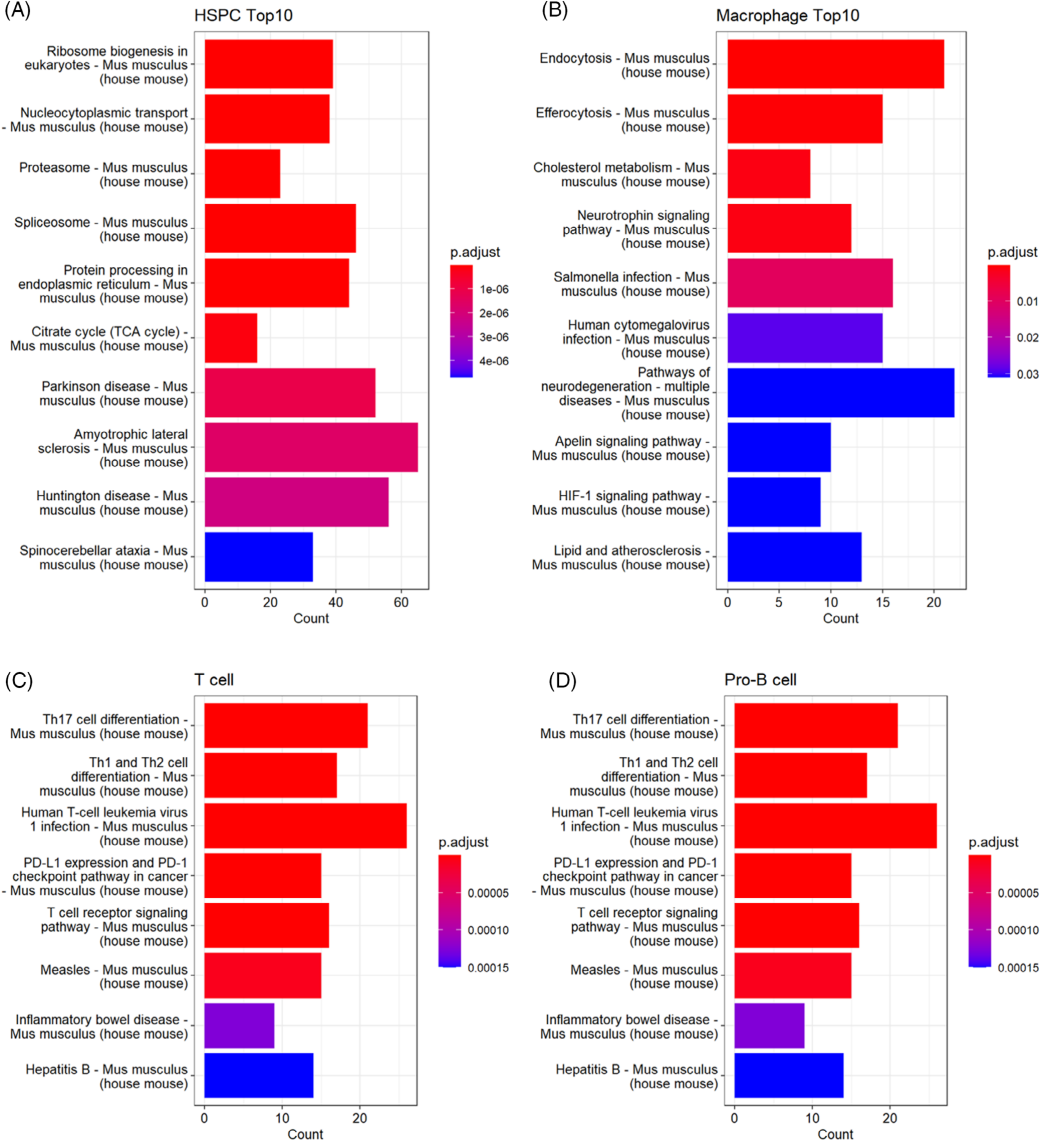
KEGG enrichment revealed the cell‐type response pathways for (A) HSPC, (B) macrophage and the identified lymphoid populations, including (C) T cell and (D) pro‐B cell. HSPC, haematopoietic stem and progenitor cell.

Compared to the recently sorted cells, 3D culture enhances the production of lymphoid‐related cells, as shown in Figure 6. KEGG analysis was conducted to investigate the primary pathways of these distinct lymphoid populations (Figure 9). The most enriched pathways for DEGs in HSPCs comprised ‘Ribosome biogenesis in eukaryotes’, ‘Nucleocytoplasmic transport’, ‘Proteasome’ and ‘Spliceosome’, among others. In line with GO principles, the highly stimulated intracellular biogenesis pathway promotes the regeneration ability of HSPCs derived from BM (Figure 9A,B). Concerning T cells, their DEGs were charted onto 42 signalling pathways, with the highest enriched pathway being Th17 cell differentiation (Figure 9C). Th17 cells, known to express IL‐17a, are an essential subset of CD4^+^ T cells that regulate the immune response of the host.^13^ The KEGG result shows improved efficiency of T helper cell differentiation by 3D hydrogel cultivation, evidenced by pathways such as ‘Th1 and Th2 cell differentiation’. The pro‐B and B cell groups share pathways such as ‘Hematopoietic cell lineage’, ‘Epstein‐Barr virus infection’ and ‘Asthma’, implying functional similarities in immunity (Figure 9D). In summary, the functional prediction confirmed the differences in cell states within specific microenvironments. This discovery highlights a modified lineage homeostasis for myeloid and lymphoid offspring, distinct from that of primary murine HPCs ex vivo.

### 2.8 The stiffness of endosteum region promotes the activation of signalling pathways correlated with actin cytoskeleton

**FIGURE 10:**
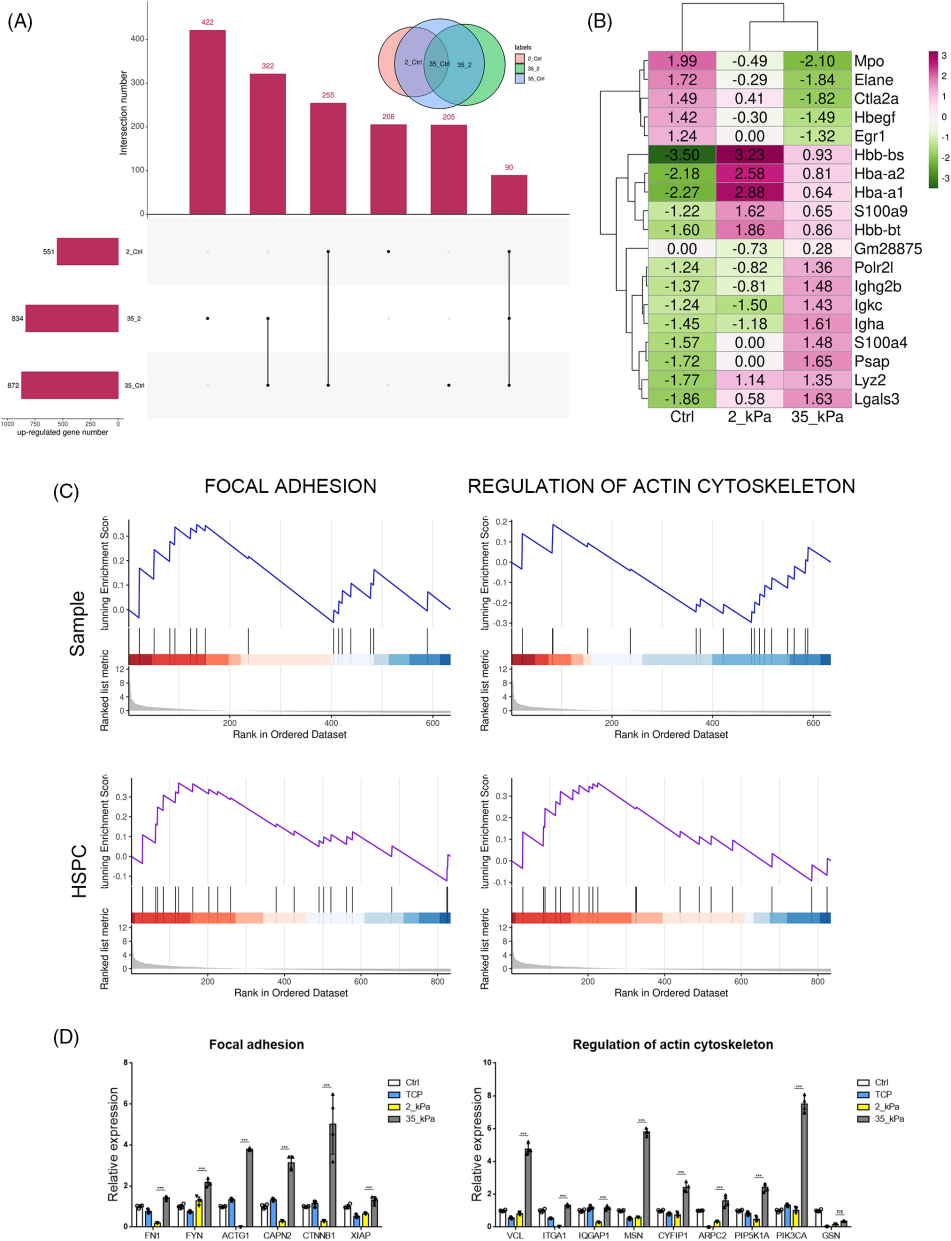
Prediction of essential pathways regulating the mechanical response and active the lineage bias programme. (A) Venn diagram reflecting DEGs associated with HPCs exposed to a comparative soft or rigid 3D environment. (B) The expression of DEGs in native bone marrow HSC and HSC cultured on different 3D environment. (C) GSEA enrichment exhibiting the activated pathways in KEGG correlation for cells embedded by hydrogels. (D) The expression levels of genes involved in terms of ‘Focal adhesion’ and ‘Regulation of actin cytoskeleton’ (*n* = 3 recipients per group from three separate experiments). DEG, differentially expressed genes; HPC, haematopoietic progenitor cell; HSC, haematopoietic stem cell.

To figure out the potential mechanical mechanisms underlying the maintenance of HSC pool and priming bias in cell differentiation, expression profiling of genetic alterations from total cell samples and the identified population of HSPC (cluster 3) were performed (Figure 10). As a result, 551 DEGs and 872 DEGs were respectively checked upregulated in cell samples from soft and stiff matrix compared to that in native BM, of which majority belong to the intersection in Figure 10A. A genetic profiling that contains 834 up‐regulated genes was evaluated on HPCs exposed to a comparative rigid 3D environment, with respect to those from a weak stiffness. According to the evaluation for cells enriched in HPSC cluster, down‐regulation of Mpo, Elane Ctla2a, Hbegf, and Egr1 were assessed. On the contrary, Hbb‐bs, Hba‐a2, Hba‐a1, and so forth. were highly expressed when cultured in vitro using hydrogels. Furthermore, the expression of Hbb‐bs, Hba‐a2, Hba‐a1, S100a9 and Hbb‐bt showed a stiffness‐dependent reduction, while Gm2885, positively expressed with the increase on matrix stiffness (Figure 10B). These analytic DEGs can provide support for the macho‐sensitivity of the 3D encapsulated HPCs, which is closely correlated with the extent of environmental rigidity.

GSEA was applied to identify activated pathways in the KEGG correlation for cells embedded in hydrogels. Relative to soft gels, alterations in gene sets in total HPCs caused by 35 kPa hydrogels have a strong positive correlation with terms such as ‘KEGG_RIBOSOME’, ‘KEGG_LYSOSOME’ and ‘KEGG_PATHWAYS_IN_CANCER’. The DEGs that respond to stiff hydrogels in the HSPC cluster were enriched to cellular pathways, namely ‘KEGG_LEISHMANIA_INFECTION’, ‘KEGG_LYSOSOME’ and ‘KEGG_MAPK_SIGNALING_PATHWAY’, as shown in Table S2. It is noteworthy that both total HPCs and HSPCs lists simultaneously exhibit several pathways that have been extensively reported to have important roles in cell‐ECM interaction, including ‘FOCAL_ADHESION’ and ‘KEGG_REGULATION_OF_ACTIN_CYTOSKELETON’ (Figure 10C,D). These findings suggest that cell adhesion to the ECM mediates mechanical interactions. The activation of DEGs by the 3D stiff hydrogel provides essential signalling landscapes through which HSPCs respond to the extracellular forces during the process of mechanotransduction. Focal adhesion and changes in the actin cytoskeleton may play a crucial role in transmitting mechanical cues to cells, which ultimately initiate the fate determination of HSCs.

## 3 CONCLUSION AND DISCUSSION

The mechanical niche, with ECM composition variations, provides extrinsic cues that regulate and transform HSC fate determinations.^14^, ^15^ Haematopoietic cells on biomaterial matrix presenting niche‐like mechanical properties such as dimensional constrains and stiffness, can minimise the diverse cues present in BM niches that control HSC fate. Herein, sub‐niche stiffness signals were created using PA hydrogels to provide elastic character of heterogenous ECM distributed from endosteum to perivascular organisations, and medullary regions in BM. Our research exhibits that matrix stiffness distinctly regulates the progenitor numbers in the HSC reservoir and the differentiation programme throughout diverse haematopoietic hierarchies. Cell surface phenotypes of LT‐HSCs, highly enriched subpopulations for long‐term repopulating HSCs, were extracted from hard substrates. Activities of later stages of HPCs, ST‐HSCs, and MPPs enable rapid reaction to compliant substrates and are suppressed with the degree of stiffness. Furthermore, myeloid specification events, including the potential for early CFU‐GEMM and CFU‐GM, as well as late CFU‐M and CFU‐E, were detected to be enhanced in a stiffness‐dependent manner. These observations are in line with selective cellular subpopulations corresponding to discrete sub‐niches. Due to the slight influence of matrix‐dimensionality variation on constituents of the HPC pool, we highlight the direct role of niche‐associated mechanical features in supporting the HSC pool.

Matrix‐stiffness are parsed out as a dominant regulator for anchoring cell adhesion to their surrounding matrix and motivating ongoing connect‐guidance based mechanical processes.^16^ In the process of mechanotransduction, an appropriate substrate stiffness is necessary to withstand the intracellular tension that cells exert. As HSCs possess weak adhesivity, a precise comprehension of the involvement of matrix stiffness for cell fate guidance is required. ScRNA‐seq has proven to be an important technique in elucidating coordination between HSCs and the niche, with the goal of explaining how HSPCs sense and respond to changes in stiffness. High‐resolution transcriptomic analysis was used to discriminate myeloid (granulocytes) cells and differentiated lymphoid (B cells and T cells) from HPCs originating from a 3D PA matrix. The data highlight the benefits of endosteum‐associated stiffness in maintaining the early phase of the HSC subset and facilitating lineage classification into macrophages, DCs, and basophils. By tracing the fate trajectory through lineage‐specific gene signatures, the dynamic changes of HPC subsets with lineage‐specific biases can be resolved, which are responsible for the production of stiffness‐induced macrophages.

Furthermore, the functional enrichment for HPCs on both soft and hard hydrogels deepen more nuanced mechanism that harness the potential to overcome current boundaries towards HSC biomechanics. The matrix stiffness enhances the activation of key DEGs involved in focal adhesion and regulation of the actin cytoskeleton. The increased levels of ITGA1, ITGB2 and ITGB7 in response to a rigid matrix imply that integrins serve as essential sensors and mediators for HSC‐ECM anchorage in the mechanism of mechanotransduction. Transcriptomic data allow us to dissect the interplay between mechanical forces acting at HSPC‐niche interfaces and the spatio‐temporal dynamics of HSPCs, which could facilitate the development of a stiffness‐based technique to engineer the functional microenvironment of HSCs and exploit it to enhance the efficacy of HSC cultures and control differentiation behaviour in 2D and 3D biomimetic niches. Furthermore, the unique biophysical mechanism of stem cells in response to their heterogeneous microenvironment is critical for the design of biomaterial analogues of bone marrow for ex vivo stem cell biomanufacturing applications.

## 4 MATERIALS AND METHODS

### 4.1 Haematopoietic progenitor cell isolation

Wide‐type female C57BL6 mice (4–7 weeks) were purchased from Laboratory Animal Center, Xi'an Jiaotong University. Primary HSC sorting procedures were performed after the approval by Lab Animal Ethics & Welfare committee of the Northwestern Polytechnical University. First, BM mononuclear cell suspension was sorted from mice femurs and tibias marrow tissue through gradient centrifugation method with FicollHypaque (density 1.084 g/mL). HPCs that were lineage negative and c‐kit positive (Lin^−^c‐kit^+^) were then isolated by magnetic activated cell sorting technology. Finally, flow cytometry was immediately performed to verify the sorting effective of Lin^−^c‐kit^+^ cell fraction from BM mononuclear cells. For HPCs culturing, IMDM medium supplemented with 10% FBS (Gibco), 50 ng/mL rm SCF, 20 ng/mL rm TPO and 20 ng/mL rm Flt‐3 L were confected to resuspend freshly sorted HPCs, and cell suspension containing the same cell count were added to 2D and 3D systems, respectively.

### 4.2 Preparation and characterisation of polyacrylamide hydrogel scaffolds

#### 4.2.1 Construction of 2D and 3D polyacrylamide hydrogels

2D hydrogel were constructed as described previously.^17^ Gel precursor solutions were prepared using polymer complexes (40% acrylamide, 2% *N*,*N* methylene‐bis‐acrylamide in water) supplemented with 10% ammonium persulphate (APS, 1:100) and *N*,*N*,*N*,*N*‐tetramethylethylenediamine (TEMED, 1:1000). The different ratio of acrylamide and methylene‐bis‐acrylamide were applied for regulate stiffness and porosity of PA hydrogels. 50 μL of the complexes was then placed on a microscopy slide treated with dichloromethylsilane, and was covered by an activated coverslip functionalised with 2% 3‐(trimethoxysilyl) propyl methacrylate to enhance its covalent attachment with hydrogels. Once gelation, the hydrogel was removed to a 6‐well plate, washed with 4‐(2‐hydroxyethyl)‐1‐piperazine ethanesulfonic acid (HEPES, 50 nM), and gel surfaces were incubated with sulphosuccinimidyl‐6‐(4′‐azido‐2′‐nitrophenylamino) hexanoate (sulfo‐SANPAH, 1 mg/mL) under UV illumination (365 nm). Finally, hydrogels were functionalised with collagen I (0.1 mg/mL) for 30 min at room temperature to provide ligands facilitating cell adhesion. Following the above procedure, we used NaCl to create a porous architecture on PA hydrogels using a salt leaching technology based on NaCl crystals as a porogen. We presented 3D porous hydrogels with NaCl crystals in the hydrogel precursor solution,^18^ which have adjustable mechanical properties and equivalent pore sizes. This provides a natural tissue microenvironment required for cell support in vitro. The NaCl crystals were ground in a mortar and then filtered through cell strainers with pore sizes of 200 μm and 100 μm (Corning). In the initial experiments, only crystals with sizes below 200 μm were collected. Subsequently, the crystals with sizes below 100 μm were removed by filtering them through the 100 μm cell strainer, and only the crystals with sizes between 100 and 200 μm were used. Polymer complexes containing acrylamide and *N*,*N* methylene‐bis‐acrylamide were prepared using a saturated aqueous NaCl solution. 100 mg of the gel precursor solution was mixed with 400 mg of size‐collected NaCl crystals. APS and TEMED were quickly added and mixed, resulting in rapid polymerisation and gelation.

#### 4.2.2 Mechanical characterisation of polyacrylamide hydrogels

Atomic force microscopy (AFM) was used to quantify the Young's modulus (stiffness) of the PA hydrogels. Samples were balanced in PBS and measured at parameter of a velocity of 2 μm/s until a 2 nN trigger force. Then results were analysed by SPIP 6.3.3 software (Image Metrology, Denmark) to determine stiffness of the PA hydrogels comprising different ratios of acrylamide and methylene‐bis‐acrylamide.

#### 4.2.3 Water absorption of PA hydrogels

The water absorption of hydrogels was measured by immersing lyophilised gels in PBS buffer (pH = 7.4) at 37°C, and then measuring their weights after various incubation times with an analytical balance until a constant weight was observed.

#### 4.2.4 The microstructures of PA hydrogels

PA hydrogels and porous gels were lyophilised and imaged via scanning electron microscopy (SEM, Tescan, Czech Republic) at 10 kV after being sputtered with platinum. Image J 1.52v software was used to analyse the pore structure characteristics of hydrogels using the obtained SEM images. The sum of the areas of the pores was divided by the total exposed sample areas to obtain the percent porosity.

### 4.3 Observation of cell morphology

A confocal microscopy was used for analysing cell spreading on different culture conditions. Images were imported to Image J 1.52v software, and individual cells were randomly outlined for the quantifications of perimeter and spread area. CSI represents a dimensionless assess of a cell circularity, and was measured as (4·π·(cell area)·(cell perimeter)^−2^), where 0 is the theoretical minimum for a cell infinitely elongated in one direction and 1 indicates a perfectly circular cell.^4^

### 4.4 Dynamic and static micron‐indentation

The dynamic creep response and static mechanical properties of all the cells were investigated using an AFM (MFP‐3D Infinity, Asylum Research, Oxford Instruments, USA). A spherical probe (CP‐PNPL‐SiO, NanoAndMore, Watsonville, CA, USA) with a diameter of 2 μm silicon dioxide sphere was employed for all the studies. The spring constant of the cantilever (*k*) was ~0.08 N/m and the resonant frequency (*f*) was ~17 kHz. First, the cells were cultured on gels with different elastic stiffnesses as substrates, and the cells were cultured overnight before mechanical measurements. Then, the cells were transferred to a liquid chamber for AFM analysis. Prior to the investigation, the AFM probe was immersed in the culture medial for a 15 min equilibration period. After that, the laser deflection sensitivity and spring constant were calibrated respectively. For the mechanical characterisation of each cell, the probe was precisely positioned over the central region of a single cell. To examine the dynamic mechanical response of cells, the probe was driven into the cell at a speed of 6 μm/s and held at a contact with a constant load for 10 s once the predefined force (800 pN) was reached. This allows for the study of the creep displacement of individual cells during the dwell period. The probe was subsequently retreated and maintained at the same position above the cell for an additional 10 s before the following static indentation. For the static mechanical characterisation, at the same location where the dynamic mechanical study was conducted, the sphere was brought into contact with the cell and push into it at a speed of 200 nm/s until the predefined load (800 pN) was reached, which effectively eliminate the viscous contributions. To mitigate substrate effects and the influence of the hard nucleus, all indentation depths were carefully controlled to be less than 1 μm. For data analysis, we utilised the IGOR Pro software (Version 17, Asylum Research, Oxford Instruments, USA) to identify the probe‐sample contact points. Subsequently, we calculated the elastic modulus (*E*) using the Hertz model for a spherical probe:

(4) $$E=\frac{3F\left(1-v^{2}\right)}{4\sqrt{R}}h^{3/2}$$

where *F* is the applied force, *v* is the Poisson's ratio and the cells were assumed to be incompressible at a value of 0.5 and *R* is the radius of the spherical probe (tip radius = 1 μm in this study).

### 4.5 Cell viability assays

Cell Counting Kit‐8 (CCK‐8) (Dojindo, Japan) kit was used to estimate the cell proliferative activity after hydrogel culture. Cell medium mixed with CCK‐8 solution was incubated for a fixed length of time, and the relevant absorbance at 450 nm was tested with a microplate reader.

### 4.6 Flow cytometry

A FACSCalibur system (BD Biosciences) was used for cell phenotype analysis. Cell suspensions were incubated in PBS at 4°C with combinations of appropriate antibodies. LSK (Lin^−^Sca‐1^+^c‐kit^+^) cells were stained with a cocktail of FITC‐conjugated lineage (Lin) antibodies (CD3 (17A2), CD45R (B220), CD11b, TER‐119 (TER‐119), Ly‐G6 (Gr‐1)), Alexa Fluor 700 conjugated c‐Kit (CD117) and PE‐conjugated Sca‐1. LT‐HSCs, ST‐HSCs, and MPPs were stained with a cocktail of Brilliant Violet 421‐conjugated CD135 and APC‐conjugated CD34 from LSK cells. BM myeloid cells and lymphoid cells were stained with a FITC‐anti CD11b monoclonal antibody (M1/70) and a PE‐anti CD19 monoclonal antibody (1D3). All antibodies were purchased from eBioscience or Biolegend. FACS data were analysed using FlowJo software Version 7.6.1 (TreeStar).

### 4.7 Colony‐forming unit assays

To compare the multi‐lineage potential of HPCs from different culture conditions, after accurate cell counting, cells were harvested and resuspended in methylcellulose medium (M3434, stem cell technology, Canada) and incubated at 37°C for 7 days. Different colonies were enumerated using an inverted microscope, according to the examples of the manufacturer's guideline.

### 4.8 Procedure of scRNA‐sequencing

ScRNA‐seq was performed based on the 10 × Genomics single‐cell RNA sequencing platform (10 × Genomics, Pleasanton, CA, USA). Briefly, cells were washed immediately after the hydrogel digestion. Live cells were captured by MACS with a Dead Cell Removal Kit (Miltenyi Biotec, Bergisch Gladbach, Germany) according to the recommended protocol. Cell viability (>95%) were determined twice using a haemocytometer (TC20, Bio‐Rad, Hercules, CA, USA). Droplet‐based single‐cell partitioning and complementary DNA libraries were prepared using a Chromium Single Cell 3′ GEM, Library & Gel Bead Kitq v3 (10 × Genomics); cDNA was purified using a SPRIselect Reagent Kit (Beckman Coulter). The constructed libraries were sequenced on an Illumina HiSeq X Ten sequencer (Illumina, San Diego, CA, USA) and pair‐ended 150 bp (PE150) reads were generated for downstream analysis.

### 4.9 Bioinformatic analysis for scRNA datasets

The Cell Ranger Single‐Cell Software Suite (https://support.10xgenomics.com/single‐cell‐gene‐expression/software/overview/welcome) was used to align reads, generate feature‐barcode matrices, and estimate raw gene counts. The STAR software was used for alignment (with reference genome mm10/GRCm38) of the cDNA insert sequence. Downstream analyses including quality filtering, normalisation, dimensional reduction, cell clustering and the identification of differentially expressed genes (DEGs) were performed with the R package Seurat (version 3.2.0). Briefly, cells with expression of <500 or > 6000 genes or a mitochondrial gene expression >5% of the total gene expression were removed. After filtering unwanted cells, the dataset was normalised by the total expression, multiplied by a scale factor of 10,000, and log‐transformed the result. Downstream unsupervised clustering and UMAP analysis were performed based on the statistically significant principal components with the Seurat FindClusters function (resolution = 0.8). Marker genes for each cluster were determined with the Wilcoxon rank‐sum test by the Seurat FindAllMarkers function (genes with Log Fold Change threshold above 0.25 were identified as DEG, top 10 DEGs are shown in Supplementary Table S1). For the cell type identification, we annotated clusters on the basis of DEGs in each cluster using marker genes in Mouse Cell Atlas (http://bis.zju.edu.cn/MCA/index.html) and Cell Marker Database (http://bio-bigdata.hrbmu.edu.cn/CellMarker/).

Gene Ontology (GO, http://geneontology.org/) enrichments as well as Kyoto Encyclopedia of Genes and Genomes (KEGG, http://www.kegg.jp/) pathway annotations were analysed using positive markers of target clusters, through the corresponding databases of Carlson M (2019). org. Mm.eg.db, and STRINGI, respectively. GO and KEGG terms with a *p* value <0.05 were considered to be significantly enriched.

To analyse the dynamic trajectory development of HPCs, we used package Monocle 2.0 and Monocle 3.0 to organise cells in pseudo developmental timeline, followed by optional statistical tests to find genes which varied in expression over those trajectories. The intercellular communication between defined clusters were systematically inferred and visualised using the Celltalker package, which is characterised by a comprehensive signalling molecule interaction database including multimeric ligand‐receptor pairs, soluble agonists and antagonists, as well as stimulatory and inhibitory co‐ligands and co‐receptors.

### 4.10 Quantitative RT‐PCR

Total RNA of cells harvested from different scaffolds was separated using TRIzol reagent (Invitrogen). cDNA was obtained by inverse transcription using GoScript™ Reverse Transcriptase (Promega) and acted as template for Real‐time PCR using iQ™ SYBR® Green Supermix (Bio‐rad). Gapdh was used as a standard gene in the gene expression comparisons.

### 4.11 Statistical analysis

Triplicate experiments were performed and statistical analyses were performed using GraphPad Prism 9.0. We entered replicate values and stacked them in columns. Each bar graph showed data points. Immunostained liver sections were quantified using ImageJ to quantify positive area expression. Comparisons between two groups were made using an unpaired t‐test. Results were presented as mean and standard error of the mean (SEM). Statistical significance was indicated at the level of *p < 0.05; **p < 0.01; ***p < 0.001. All experiments were repeated independently with at least three mice per experimental group. The exact number of data and images used for each experiment was given in the legends.

## AUTHOR CONTRIBUTIONS

**Guolin Shi**: Conceptualisation; methodology; investigation; writing—original draft; writing—review & editing. **Zhuo Chang**: Methodology; investigation. **Pan Zhang**: Data curation; visualisation. **Xiaohang Zou**: Conceptualisation; methodology. **Xinmin Zheng**: Conceptualisation; methodology. **Xiru Liu**: Conceptualisation; methodology. **Jinxiao Yan**: Methodology; data curation. **Huiyun Xu**: Conceptualisation; methodology. **Zhenhao Tian**: Conceptualisation; methodology. **Nu Zhang**: Methodology; data curation. **Ning Cui**: Methodology; data curation. **Leming Sun**: Data curation; visualisation. **Guangkui Xu**: Resources; language modification. **Hui Yang**: Writing—review & editing; supervision; funding acquisition.

## CONFLICT OF INTEREST STATEMENT

The authors declare that they have no known competing financial interests or personal relationships that could have appeared to influence the work reported in this paper.

## Supporting information


**TABLE S1.** Differentially expressed genes of the haematopoietic stem and progenitor cells (top 10).

**TABLE S2.** GSEA enrichment for the cells embedded in soft and stiff hydrogels.

## Data Availability

The data that supports the findings of this study are available in the supplementary material of this article.

## References

[1] Vining KH , Mooney DJ . Mechanical forces direct stem cell behaviour in development and regeneration. Nat Rev Mol Cell Biol. 2017;18:728‐742. 29115301 10.1038/nrm.2017.108 PMC5803560

[2] Seita J , Weissman IL . Hematopoietic stem cell: self‐renewal versus differentiation. Wiley Interdiscip Rev Syst Biol Med. 2010;2:640‐653. 20890962 10.1002/wsbm.86 PMC2950323

[3] Choi JS , Mahadik BP , Harley BA . Engineering the hematopoietic stem cell niche: Frontiers in biomaterial science. Biotechnol J. 2015;10:1529‐1545. 26356030 10.1002/biot.201400758 PMC4724421

[4] Choi JS , Harley BA . Marrow‐inspired matrix cues rapidly affect early fate decisions of hematopoietic stem and progenitor cells. Sci Adv. 2017;3:e1600455. 28070554 10.1126/sciadv.1600455 PMC5218514

[5] Zhang P , Zhang C , Li J , Han J , Liu X , Yang H . The physical microenvironment of hematopoietic stem cells and its emerging roles in engineering applications. Stem Cell Res Ther. 2019;10:327. 31744536 10.1186/s13287-019-1422-7 PMC6862744

[6] Saçma M , Pospiech J , Bogeska R , et al. Haematopoietic stem cells in perisinusoidal niches are protected from ageing. Nat Cell Biol. 2019;21:1309‐1320. 31685996 10.1038/s41556-019-0418-y

[7] Lee‐Thedieck C , Rauch N , Fiammengo R , Klein G , Spatz JP . Impact of substrate elasticity on human hematopoietic stem and progenitor cell adhesion and motility. J Cell Sci. 2012;125:3765‐3775. 22553208 10.1242/jcs.095596

[8] Leiva O , Leon C , Kah Ng S , Mangin P , Gachet C , Ravid K . The role of extracellular matrix stiffness in megakaryocyte and platelet development and function. Am J Hematol. 2018;93:430‐441. 29247535 10.1002/ajh.25008 PMC5803306

[9] Guinard I , Nguyen T , Brassard‐Jollive N , et al. Matrix stiffness controls megakaryocyte adhesion, fibronectin fibrillogenesis, and proplatelet formation through Itgβ3. Blood Adv. 2023;7:4003‐4018. 37171626 10.1182/bloodadvances.2022008680 PMC10410137

[10] Chen X , Zhao J , Gu C , et al. Med23 serves as a gatekeeper of the myeloid potential of hematopoietic stem cells. Nat Commun. 2018;9:3746. 30218073 10.1038/s41467-018-06282-2 PMC6138688

[11] Grover A , Sanjuan‐Pla A , Thongjuea S , et al. Single‐cell RNA sequencing reveals molecular and functional platelet bias of aged haematopoietic stem cells. Nat Commun. 2016;7:11075. 27009448 10.1038/ncomms11075 PMC4820843

[12] Singh V , Davidson AC , Hume PJ , Humphreys D , Koronakis V . Arf GTPase interplay with Rho GTPases in regulation of the actin cytoskeleton. Small GTPases. 2019;10:411‐418. 28524754 10.1080/21541248.2017.1329691 PMC6748364

[13] Hang S , Paik D , Yao L , et al. Bile acid metabolites control T(H)17 and T(reg) cell differentiation. Nature. 2019;576:143‐148. 31776512 10.1038/s41586-019-1785-z PMC6949019

[14] Ilin Y , Choi JS , Harley BA , Kraft ML . Identifying states along the hematopoietic stem cell differentiation hierarchy with single cell specificity via Raman spectroscopy. Anal Chem. 2015;87:11317‐11324. 26496164 10.1021/acs.analchem.5b02537 PMC4687963

[15] Zhang P , Xu L , Gao J , et al. 3D collagen matrices modulate the transcriptional trajectory of bone marrow hematopoietic progenitors into macrophage lineage commitment. Bioact Mater. 2022;10:255‐268. 34901544 10.1016/j.bioactmat.2021.08.032 PMC8636680

[16] Leclech C , Barakat AI . Is there a universal mechanism of cell alignment in response to substrate topography? Cytoskeleton. 2021;78:284‐292. 33843154 10.1002/cm.21661

[17] Wen JH , Vincent LG , Fuhrmann A , et al. Interplay of matrix stiffness and protein tethering in stem cell differentiation. Nat Mater. 2014;13:979‐987. 25108614 10.1038/nmat4051 PMC4172528

[18] Raic A , Rödling L , Kalbacher H , Lee‐Thedieck C . Biomimetic macroporous PEG hydrogels as 3D scaffolds for the multiplication of human hematopoietic stem and progenitor cells. Biomaterials. 2014;35:929‐940. 24176196 10.1016/j.biomaterials.2013.10.038

